# Histamine and histamine receptor H1 (HRH1) axis: new target for enhancing immunotherapy response

**DOI:** 10.1186/s43556-022-00073-4

**Published:** 2022-04-05

**Authors:** Ziyi Bai, Jiayuan Ai, Xiawei Wei

**Affiliations:** Laboratory of Aging Research and Cancer Drug Target, State Key Laboratory of Biotherapy, National Clinical Research Center for Geriatrics, West China Hospital, Sichuan University, No. 17, Block 3, Southern Renmin Road, Chengdu, Sichuan 610041 People’s Republic of China

Li and colleagues recently demonstrated in *Cancer Cell* that the histamine receptor H1 (HRH1) axis could serve both as therapeutic targets for enhancing immunotherapy response and predictive biomarker of T cell exhaustion and therapeutic effectiveness in cancer immunotherapy [1] **(**Fig. 1**)**.

**Fig. 1:**
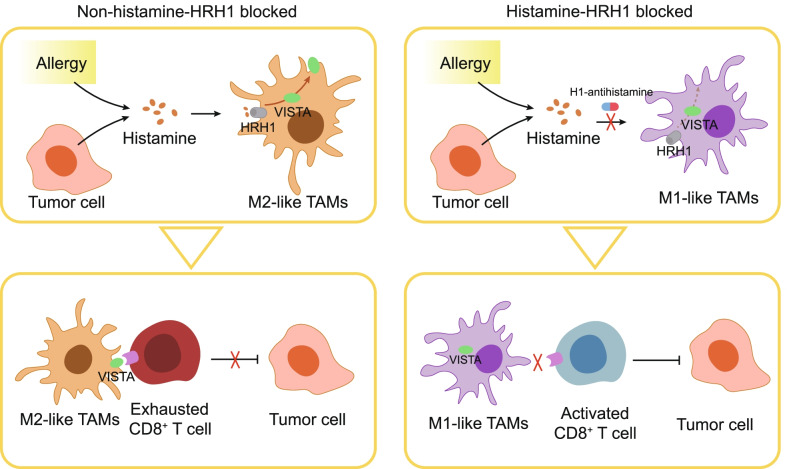
The impact of Histamine binding to macrophages histamine receptor H1 (HRH1) on the tumor microenvironment. Tumor-derived or allergy-released histamine bind to HRH1 educating macrophages toward the M2 activation status and promoting V-domain Ig suppressor of T cell activation (VISTA) membrane localization. Conversely, blocking HRH1 elicit the polarization of M1 macrophages and reduce the expression of VISTA in the cell membrane, promoting CD8^+^ T cell function and inhibiting tumor growth. TAMs, tumor-associated macrophages

Histamine, a metabolite from catalyzation of histidine by the enzyme L-histidine decarboxylase, is located throughout the entire organism, acting its biological effects through four subtypes of G-protein coupled receptors (HRH1-HRH4). Over the past 20 years, a large number of studies demonstrated the pathophysiological roles of histamine in cancer, and there is now overwhelming evidence supporting the effects of histamine receptors on tumor progression [2]. Among them, HRH1 is the first identified important targets of receptor subtypes for clinical application, as its antagonists are commonly used in the treatment of allergic symptoms. However, various mechanisms of histamine and HRH1 in cancer development have remained largely elusive to date. Li et al. now found in a retrospective analysis that treatment with HRH1-specific antihistamines during immune checkpoint blockade (ICB) treatment was associated with significantly improved clinical outcomes. These findings suggested that HRH1-antihistamines may augment antit-umor immunity, which raised an interesting question: how do HRH1-specific antihistamines affect anti-tumor immunity?

Li et al. found that HRH1 expression was associated with higher tumor immune dysfunction scores and poor survival in most cancer types. Further analysis in tumor-derived cell lines showed that HRH1 expression was positively correlated with tumor-associated macrophages (TAMs). In particular, HRH1 was most strongly associated with immunosuppressed M2-like macrophages among a variety of cell types of human tumor microenvironment. Next, the authors explored the specific functional programs of the histamine-HRH1 axis in macrophages. Here, wild-type (WT) and HRH1 knockout (HRH1^−/−^) bone marrow derived macrophages were isolated and cultured in tumor-cell-conditioned medium (TCM). The addition of HRH1^−/−^ or antihistamine H1-antihistamine fexofenadine (FEXO) eliminated TAM-mediated T cell inhibition by promoting T cell proliferation and upregulating cytotoxic immune cells and cytolytic effector molecules, enhancing the ability to kill tumor cells. Meanwhile, the authors transplanted WT or HRH1^−/−^ macrophages with various types of cancer cells into recipient mice, and finally determined that activation of HRH1 in macrophages inhibited CD8^+^ T cell response and promoted tumor growth.

The authors then investigated whether HRH1 on macrophages induced T cell exhaustion via regulating co-stimulatory or co-inhibitory receptors on T cells. As expected, they identified known inhibitory molecules V-domain Ig suppressor of T cell activation (VISTA) was a major HRH1 downstream mediator of T cell exhaustion. Notably, blocking HRH1 significantly reduced VISTA membrane expression in macrophages, but there was no significant change in VISTA total protein expression. By blocking Calcium (Ca^2+^) flux, they confirmed that HRH1-modulated Ca^2+^ release was critical for VISTA membrane localization.

To elucidate which downstream HRH1 signals may lead to immunosuppressive phenotypes in macrophages, the authors performed transcriptomic characterization analysis of WT and HRH1^−/−^ macrophages treated by TCM. Compared with WT macrophages, HRH1^−/−^ macrophages exhibited lower expressions of genes associated with the M2-like phenotype (*C1QB, C1QC*) but higher M1 polarization-related genes (*CXCL10, CD40*). Single-cell RNA sequencing further identified the effect of HRH1 blockade on macrophage phenotype and TME in vivo that HRH1-activated macrophages were polarized to an M2-like immunosuppressive phenotype, and reduced cytotoxic immune cells. In addition, these results were confirmed by associations with cell markers at the single-cell level, suggesting that blocking HRH1 could remodel the transcriptome characteristics of immune cells.

Li et al. essentially studied the effect of HRH1 blockade on immunotherapy using a repertoire of WT/mutant murine tumor models. In general, it was reported that (i) non-responding tumors had higher HRH1 and VISTA expression on TAMs than partially responding tumors under anti programmed cell death protein 1 (PD-1) treatment, (ii) inhibition of HRH1 enhanced the anti-tumor immunity of programmed death-ligand 1 (PD-L1) and cytotoxic T-lymphocyte-associated antigen 4 (CTLA-4) blockade, (iii) FEXO in combination with ICB (anti-PD-1/anti-CTLA-4) achieved the higher therapeutic effect compared to FEXO or ICB alone. Of note, VISTA-blocking antibodies are undergoing clinical trials for antitumor efficacy [3]. The authors found that FEXO had similar antitumor activity to VISTA antibody, and even ICB treatment with FEXO was more effective than ICB treatment with VISTA in prolongating mouse survival and promoted M1-like polarization of macrophages.

Since allergic reactions release large amounts of histamine [4], a key question now is whether anaphylaxis also affects antitumor immunity and immunotherapy responses. Therefore, an allergic airway disease mice model with transplanted tumor cells were used. In short, allergy promoted tumor growth and induced immunotherapy resistance through the histamine-HRH1 axis. Finally, by comparing plasma histamine levels in a group of cancer patients before treatment with anti-PD-1, the authors showed that patients with low plasma histamine levels had significantly increased overall response rates and disease control rates. These results supported the presence of hypersensitivity to histamine in plasma, impairing the antitumor immune response of cancer patients and leading to their adverse response to immunotherapy.

Altogether, these data indicated that blocking the binding of tumor-derived or allergy-released histamine to HRH1 on TAMs enhanced cytotoxic T cell function and alleviated immunosuppression of TME. Of note, M2 macrophages are similar in phenotype to TAMs, promoting tumor growth and metastasis, while blocking HRH1 reduced M2-like macrophages cell composition [5]. However, it is still not clear how activation of HRH1 signaling influences the macrophages polarization.

Of note, it remains to be determined how antihistamines regulate other downstream immune effectors other than VISTA, as antihistamines combined with ICB elicited stronger antitumor responses than anti-VISTA antibodies combined with ICB. Unfortunately, allergy records of patients who received antihistamine therapy before ICB treatment were not included in this study, so further clinical studies are needed to prospectively examine the effect of H1-antihistamine adjuvant therapy in augmenting the response to cancer immunotherapy.

## Data Availability

Not applicable.

## Abbreviations

HRH1: Histamine receptor H1
ICB: Immune checkpoint blockade
TAMs: Tumor-associated macrophages
WT: Wild-type
HRH1^**−**^^**/**^^**−**^: HRH1 knockout
TCM: Tumor-cell-conditioned medium
FEXO: Fexofenadine
VISTA: V-domain Ig suppressor of T cell activation
Ca^2^^**+**^: Calcium
PD-1: Programmed cell death protein 1
PD-L1: Programmed death-ligand 1
CTLA-4: Cytotoxic T-lymphocyte-associated antigen 4
